# Airway management by physician-staffed Helicopter Emergency Medical Services – a prospective, multicentre, observational study of 2,327 patients

**DOI:** 10.1186/s13049-015-0136-9

**Published:** 2015-08-07

**Authors:** Geir Arne Sunde, Jon-Kenneth Heltne, David Lockey, Brian Burns, Mårten Sandberg, Knut Fredriksen, Karl Ove Hufthammer, Akos Soti, Richard Lyon, Helena Jäntti, Antti Kämäräinen, Bjørn Ole Reid, Tom Silfvast, Falko Harm, Stephen J.M. Sollid, for The Airport Study Group

**Affiliations:** Norwegian Air Ambulance Foundation, Drøbak, Norway; Department of Anaesthesia and Intensive Care, Haukeland University Hospital, Bergen, Norway; Department of Health Sciences, University of Stavanger, Stavanger, Norway; Department of Medical Sciences, University of Bergen, Bergen, Norway; Institute of Prehospital Care, London’s Air Ambulance, Bartshealth NHS Trust, London, UK; Sydney HEMS, Ambulance Service of NSW, Sydney, Australia; Sydney Medical School, University of Sydney, Sydney, Australia; Air Ambulance Department, Oslo University Hospital, Oslo, Norway; Faculty of Medicine, University of Oslo, Oslo, Norway; UiT - The Arctic University of Norway, Tromsø, Norway; The University Hospital of North Norway, Tromsø, Norway; Centre for Clinical Research, Haukeland University Hospital, Bergen, Norway; Hungarian Air Ambulance Nonprofit Ltd., Budaors, Hungary; Emergency Medicine Research Group, Edinburgh, UK; Kent, Surrey & Sussex Air Ambulance Trust, Marden, UK; Centre for Prehospital Emergency Care, Kuopio University Hospital, Kuopio, Finland; Emergency Medical Services, Tampere University Hospital, Tampere, Finland; Department of Emergency Medicine and Prehospital Services, St. Olavs Hospital, Trondheim, Norway; Emergency Medical Services, Helsinki University Hospital, Helsinki, Finland; University of Helsinki, Helsinki, Finland; Department for Anaesthesia, Surgical Intensive Care, Prehospital Emergency Medicine and Pain Therapy, University Hospital Basel, Basel, Switzerland

**Keywords:** Advanced trauma life support, Airway management, Emergency medical services, Intubation, Out-of-hospital cardiac arrest

## Abstract

**Background:**

Despite numerous studies on prehospital airway management, results are difficult to compare due to inconsistent or heterogeneous data. The objective of this study was to assess advanced airway management from international physician-staffed helicopter emergency medical services.

**Methods:**

We collected airway data from 21 helicopter emergency medical services in Australia, England, Finland, Hungary, Norway and Switzerland over a 12-month period. A uniform Utstein-style airway template was used for collecting data.

**Results:**

The participating services attended 14,703 patients on primary missions during the study period, and 2,327 (16 %) required advanced prehospital airway interventions. Of these, tracheal intubation was attempted in 92 % of the cases. The rest were managed with supraglottic airway devices (5 %), bag-valve-mask ventilation (2 %) or continuous positive airway pressure (0.2 %). Intubation failure rates were 14.5 % (first-attempt) and 1.2 % (overall). Cardiac arrest patients showed significantly higher first-attempt intubation failure rates (odds ratio: 2.0; 95 % CI: 1.5-2.6; *p* < 0.001) compared to non-cardiac arrest patients. Complications were recorded in 13 %, with recognised oesophageal intubation being the most frequent (25 % of all patients with complications). For non-cardiac arrest patients, important risk predictors for first-attempt failure were patient age (a non-linear association) and administration of sedatives (reduced failure risk). The patient’s sex, provider’s intubation experience, trauma type (patient category), indication for airway intervention and use of neuromuscular blocking agents were not risk factors for first-attempt intubation failure.

**Conclusions:**

Advanced airway management in physician-staffed prehospital services was performed frequently, with high intubation success rates and low complication rates overall. However, cardiac arrest patients showed significantly higher first-attempt failure rates compared to non-cardiac arrest patients. All failed intubations were handled successfully with a rescue device or surgical airway.

**Trial registration:**

Study registration: www.clinicaltrials.govNCT01502111. Registered 22 December 2011.

## Background

The frequency of prehospital tracheal intubation (TI) failure and adverse events may be influenced by the unique challenges encountered in the field [1, 2]. Unfortunately, despite high rates of reported unanticipated difficult laryngoscopy and airway related complications from some services, there is a shortage of high-quality data to assess the efficacy and benefits of prehospital TI [2–5]. Despite the publication of numerous prehospital airway studies, inconsistent and imprecise reporting of data persists [3].

Recognition that TI is a ‘complex intervention’ which needs to be performed by an experienced practitioner, emphasises the importance of international standards for documentation and reporting of airway management [3]. An Utstein-style template for uniform reporting of data from prehospital advanced airway management has therefore been developed [6]. To our knowledge, consistent reporting standards have never been implemented across international physician-staffed helicopter emergency medical services (HEMS). The objective of this study was to assess airway management from international HEMS that provide emergency airway interventions, using the uniform Utstein-style airway template for collecting data.

## Methods

This study was designed as an international prospective multicentre observational study, collecting data on airway management in critically ill or injured patients, and in patients with cardiac arrest (CA) of traumatic or medical cause, according to the Utstein-style airway template [6]. Necessary ethical and institutional approvals were acquired prior to patient enrolment (see the acknowledgements section for full details), and the study was registered in www.clinicaltrials.gov (NCT01502111). Need for written consent was waived, as the study collected anonymised data. Study results are presented according to the STROBE guidelines for observational studies [7].

Twenty-one physician-staffed HEMS from Australia (Greater Sydney Area HEMS), England (London’s Air Ambulance and Kent Surrey Sussex HEMS), Finland (Kuopio, Vantaa and Tampere), Hungary (Budaörs, Balatonfüred, Sármellék, Pécs, Szentes, Debrecen and Miskolc), Norway (Lørenskog, Bergen, Stavanger, Tromsø, Trondheim, Ål and Arendal) and Switzerland (REGA-Basel) participated. All HEMS were staffed by pilot, flight paramedic and physician. Physicians were mainly anaesthesiologists or emergency physicians at specialist level or in speciality training fulfilling mandatory minimum service requirements (e.g. in anaesthesia) for HEMS duty. Twenty of twenty-one participating services attended both trauma and medical cases, while one service (London Air Ambulance) attended predominately trauma cases. All services were capable of providing medication facilitated TI or surgical airway on-scene. Difficult airway and rapid sequence induction (RSI) protocols were part of local standard operating procedures (SOP) [8]. Service-specific anaesthetic agents, sedatives, analgesics and neuromuscular blocking agents (NMBA) (e.g. suxamethonium, rocuronium, vecuronium, or cisatracurium) were available to facilitate airway management.

All patients that received advanced airway management on HEMS primary missions (response to the scene of accident or illness outside the hospital) during the study period were included. Patients receiving airway management during secondary missions (inter-hospital transfers) were excluded. Data collection commenced on 1st January 2012 for the majority of centres and was concluded on 15th March 2013 for the last centres. The participating centres collected data over a 12-month period, except for two centres (Kent Surrey Sussex HEMS, England; and REGA-Basel, Switzerland) that participated for nine and six months, respectively.

Advanced airway management included insertion of airway devices (e.g. TI or supraglottic airway device (SAD)), airway interventions (e.g. surgical airway), and/or the administration of ventilatory assistance (e.g. bag-valve-mask (BVM) ventilation or mechanical ventilation). A TI attempt was defined as attempted laryngoscopy with the intent to intubate. Successful TI was defined as a tracheal tube verified in the trachea, usually by visual inspection, auscultation and end tidal carbon dioxide (ETCO_2_) measurement. Physicians were asked to register data from the time of emergency call to the Emergency Dispatch Centre to time of patient admission to hospital or death on-scene, and to record complications that occurred during or immediately after airway management. Data were collected according to consensus-derived core dataset definitions, proposed and described in the Utstein-style airway template article (for details see http://www.sjtrem.com/content/17/1/58) [6]. We also collected data proposed as ‘optional’ but potentially useful. Survival data were available only for the prehospital phase, and follow-up after hospital admission was beyond the scope of this study. The use of drugs to facilitate TI was recorded. Data definitions were available to the physicians recording both on paper form and on screen as the data was being entered. Data were usually registered by attending physicians after completed mission, or at the end of the day if opportune, and entered by the physician or a project coordinator through a secure Web-based system, based on a Microsoft SharePoint® 2010 database (Microsoft Corporation, Redmond, USA) at Haukeland University Hospital, Bergen, Norway. Local project coordinators monitored patient enrolment, data quality and data capture throughout the study period.

To estimate the effect of various risk factors for first-attempt TI failure (e.g. age, sex, intubation experience, trauma type, drugs administered and the indication for airway intervention), we fitted a mixed-effect complete-case logistic regression model with HEMS centre as a random effect and the potential risk factors as fixed effects. To capture the non-linear association with age, this variable (measured in decades and centred on age 50) was included as a quadratic effect. For each potential risk factor, we also fitted similar ‘unadjusted’ models, where the effect of each risk factor was not adjusted for the other variables (but did include a random effect for HEMS centre). We prefer the assumption of homogeneity of odds ratios to one of homogeneity of relative risks [9], but also fitted a similar Poisson mixed-effects for the relative risks, as a type of sensitivity test. For this study, there was little difference between the models (results not shown).

To evaluate the predictive capabilities of our model, we used 10-fold cross validation. The dataset was randomly divided into ten equal parts. We then used nine parts to fit the model and to estimate the odds of failure for the patients in the last part. This was repeated for all ten parts, giving estimated failure odds (and risks) for each patient. The patients were then categorised into deciles based on these risks before average estimated failure risk within each decile was compared with the empirical risk (the number of failures divided by the number of patients). We used the conditional modes for the random effect in creating these estimates.

To test the association between cardiac arrest and intubation failure and other complications, we use the Cochran-Mantel-Haenszel estimators and tests, with HEMS centres as strata.

We used IBM SPSS Statistics version 21 for storing and preparing the data for statistical analysis and R version 3.1.1 for all statistical analysis [10]. The mixed effects logistic model was fitted with the R package lme4 version 1.1-7 [11].

## Results

### Overview

The participating HEMS attended 14,703 patients on primary missions during the study period, and 2,327 (16 %) required advanced prehospital airway interventions. Of these, the patient categories were medical (55 %), blunt trauma (41 %), penetrating trauma (3 %) or unknown (1 %). Twenty-eight percent died on-scene, or were pronounced dead on arrival in hospital, while 71 % survived to hospital admission, and in 1 % the primary outcome was unknown. The majority of patients (72 %) were male. Patient characteristics are shown in Table 1.

**Table 1:** Patient characteristics

Patient category	Blunt Trauma^a^		Penetrating trauma		Non-trauma^b^		All categories^c^	
Patients	953		82		1269		2327	
In Cardiac Arrest	145	15 %	34	41 %	790	62 %	980	42 %
Age								
0–5 years	22	2 %	0	0 %	43	3 %	66	3 %
6–14 years	40	4 %	3	4 %	25	2 %	68	3 %
15–29 years	239	26 %	35	43 %	66	5 %	344	15 %
30–49 years	306	33 %	28	34 %	206	17 %	544	24 %
50–69 years	224	24 %	11	13 %	533	43 %	776	34 %
+70 years	101	11 %	5	6 %	361	29 %	473	21 %
Median (range)	40 (0–95)		30 (9.5–79)		62 (0–95)		53 (0–95)	
Missing data	21		0		35		56	
Sex								
Male	702	74 %	72	88 %	879	70 %	1671	72 %
Female	248	26 %	10	12 %	376	30 %	638	28 %
Missing data	3		0		14		18	
Comorbidity (ASA-PS)								
ASA 1	555	66 %	54	73 %	233	20 %	842	41 %
ASA 2	209	25 %	16	22 %	410	35 %	635	31 %
ASA 3	68	8 %	3	4 %	415	36 %	486	23 %
ASA 4–6	6	1 %	1	1 %	99	9 %	107	5 %
Missing data	115		8		112		238	

^a^Blunt trauma, including burns and strangulation

^b^Non-trauma, including drowning and asphyxia

^c^Including 23 patients with unknown trauma category

*ASA-PS* American Society of Anesthesiologists Physical Status

Half of the TIs (52 %) were done by providers that had performed over 1,000 previous TIs, while one third were done by providers with 101–1,000 previous TIs. Tracheal intubation was attempted in 2,144 patients (92 %), with a first-attempt failure rate of 14.5 % and an overall failure rate of 1.2 %.

The remaining 183 patients were managed with SAD (67 %), BVM (30 %) or continuous positive airway pressure (CPAP) (3 %). After failed TI, all airways were handled successfully with BVM (eight cases), SAD (fifteen cases) or surgical airway (three cases). Surgical airway was performed in six patients (0.3 %), (one primary surgical airway, one after failed BVM, one after failed SAD, and three after failed TI). An additional two patients were intubated through their permanent tracheostomies.

### Cardiac arrest patients

Of the 2,327 patients, 980 (42 %) presented with CA, which was recorded as the main indication for airway intervention in 15 % of the blunt trauma cases, 41 % of the penetrating trauma cases and 62 % of the medical cases. Survival rates to hospital were 28, 18 and 40 %, respectively. The first-attempt airway intervention was BVM (37 %), SAD (20 %), TI (40 %) or unrecorded (2 %). The successful airway management was TI (84 %), SAD (13 %), BVM (2 %), surgical airway (0.4 %) or unknown (0.2 %). Of intubation attempts, 80 % were successful on the first attempt, 12 % after more than one attempt and one rescuer, 6 % after more than one attempt and multiple rescuers and 2 % were not successful. The CA patients showed significantly higher first-attempt failure rates than non-CA patients, with an odds ratio of 2.0 (95 % CI: 1.5–2.6; *p* < 0.001). In 93 % of the CA cases, no drugs were used to facilitate airway interventions (‘cold intubations’), the remaining receiving sedatives (5 %), neuromuscular blocking agents (NMBA) (6 %) and/or analgesics (4 %).

### Non-cardiac arrest patients

For the 1,347 non-CA patients (58 %), survival rates to hospital were 97 % (blunt trauma), 94 % (penetrating trauma) and 93 % (medical), respectively. The main indications for airway intervention were decreased level of consciousness (61 %), ineffective ventilation (11 %), combative or uncooperative patient (8 %), impending airway obstruction (6 %), hypoxaemia (5 %), relief of pain or distress (4 %), existing airway obstruction (2 %), other (1 %) or unknown (3 %).

The first airway intervention was TI (78 %), BVM (16 %), SAD (4 %) or unrecorded (2 %). Final successful airway management was TI (96 %), BVM (3 %), SAD (1 %), CPAP (0.4 %), surgical airway (0.1 %) or unknown (0.3 %). Of TI attempts, 89 % were successful on first attempt, 7 % after more than one attempt and one rescuer and 3 % after more than one attempt and multiple rescuers. Nine TI attempts (0.7 %) failed.

**Fig. 1:**
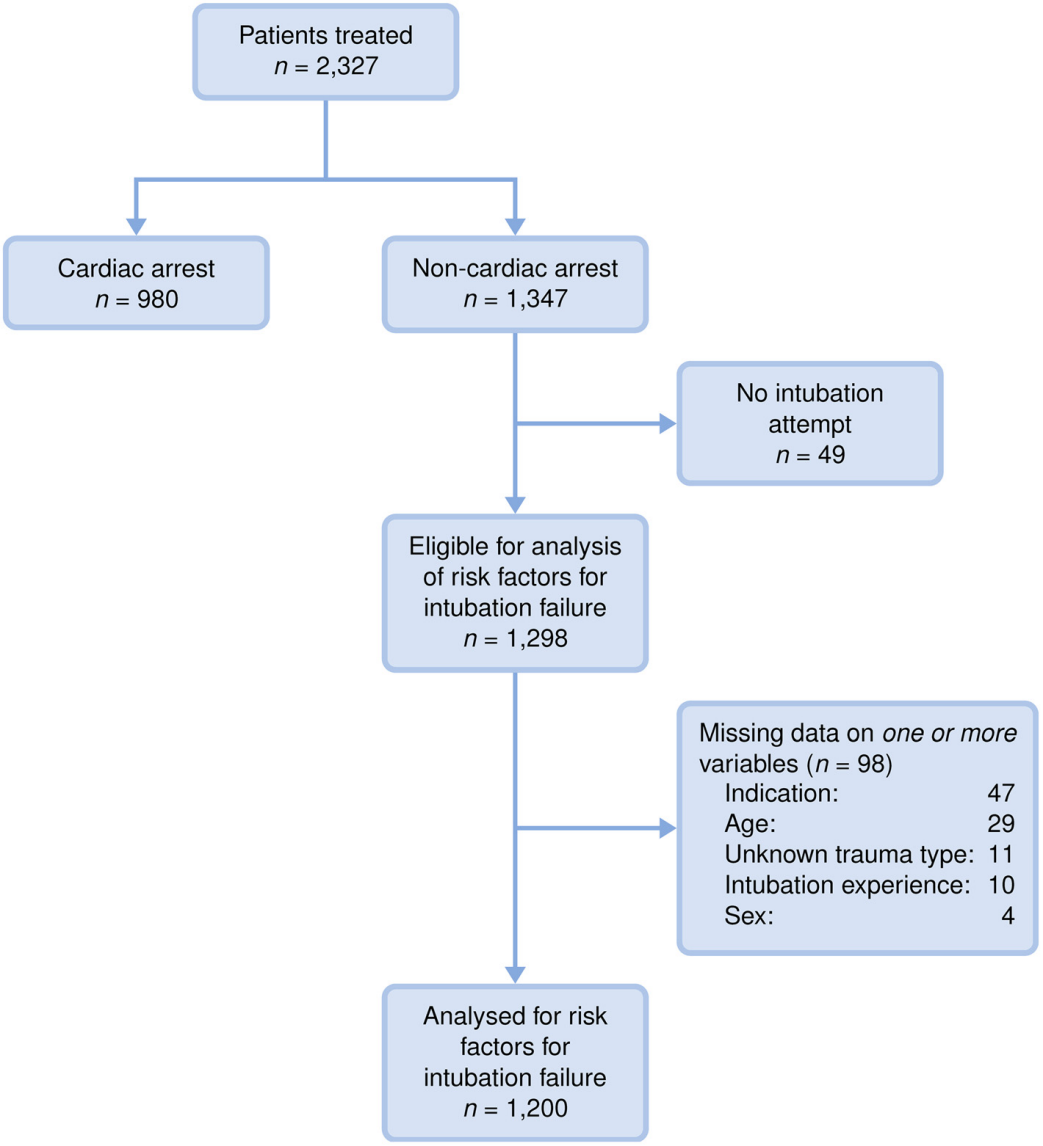
Patient flowchart

**Fig. 2:**
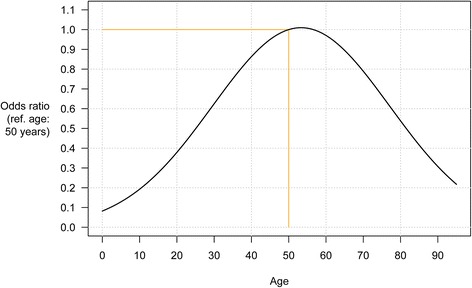
Estimated odds ratios for the effect of age on intubation failure on first attempt (ref.: 50 years)

Estimated odds ratios (with 95 % confidence intervals) for failures on the first intubation attempt are shown in Table 2. There was little missing data (7.6 % of cases – see Fig. 1), so we used a complete-case analysis. The only important risk predictors were the patient’s age and the administration of sedatives. There was a non-linear association between the patient’s age and the failure risk, with the highest risk for middle-aged patients (a peak around 53 years), and significantly lower risk for both younger and older patients; see Fig. 2. The age–risk association was similar in adjusted and unadjusted analyses. (As a sensitivity analysis, we also fitted a spline function for the age–risk association, and this turned out to be well described by the simple quadratic function presented.) No age effect was found when fitting a similar model for CA patients (results not shown). Most of the patients (88 %) received sedatives, but the ones who did not had estimated double odds of intubation failure (though this was only statistically significant in the unadjusted analysis, and borderline significant in the adjusted analysis; *p* = 0.06). The other drugs administered did not show any statistically significant relationship with first-attempt intubation failure. (We also tested the effect of combinations of drugs, with no significant effects; results not shown.) The patient’s sex, the provider’s intubation experience, the trauma type (patient category) and the indication for airway intervention did not show any association with the risk of first-attempt intubation failure for these non-CA patients. The estimated standard deviation for the random effect of HEMS sites in our model was 0.95 (95 % CI: 0.62–1.52) on the logit scale. The model seemed to have good predictive power (Table 4).

**Table 2:** Estimated odds ratios (with 95 % confidence intervals) for the risk of failure on the first intubation attempt, based on a mixed-effects logistic regression model with HEMS as a random effect (*n* = 1,200)

		Unadjusted			Adjusted		
	*n*	Odds ratio	95 % CI	*p*-value	Odds ratio	95 % CI	*p*-value
Intercept (reference odds)	1,200	0.09	(0.05–0.14)	–	0.18	(0.06–0.26)	–
Sex				0.92			0.88
Female (ref.)	355	1	–	–	1	–	–
Male	845	1.02	(0.68–1.54)	0.92	1.04	(0.67–1.57)	0.88
Age^a^				<0.001			< .001
Age_10_	1,200	1.071	(0.960–1.193)	–	1.060	(0.946–1.189)	–
Age_10_-squared	1,200	0.916	(0.873–0.961)	<0.001	0.914	(0.871–0.960)	<0.001
Provider’s previous number of intubations				0.48			0.46
> 1,000 (ref.)	587	1	–	–	1	–	–
101–1,000	514	0.99	(0.61–1.55)	0.96	0.95	(0.58–1.55)	0.84
26–100	74	1.19	(0.57–2.51)	0.64	1.24	(0.56–2.71)	0.60
11–25	12	0.23	(0.03–1.85)	0.17	0.21	(0.03–1.71)	0.14
0–10	13	1.43	(0.40–5.05)	0.58	1.08	(0.28–4.12)	0.92
Drugs administered							0.16
Sedatives	1,057	0.57	(0.34–0.97)	0.05	0.53	(0.28–1.02)	0.06
NMBA	1,110	0.67	(0.36–1.22)	0.21	0.82	(0.40–1.70)	0.60
Analgesics/opioids	858	1.11	(0.70–1.76)	0.66	1.51	(1.88–2.59)	0.14
Trauma				0.95			0.98
Non-trauma^b^ (ref.)	390	1	–	–	1	–	–
Blunt trauma^c^	763	0.93	(0.60–1.45)	0.75	1.05	(0.64–1.71)	0.84
Penetrating trauma	47	0.93	(0.31–2.82)	0.90	0.98	(0.31–3.10)	0.97
Indication				0.30			0.41
Decreased level of consciousness (ref.)	780	1	–	–	1	–	–
Hypoxemia	46	1.41	(0.56–3.57)	0.47	1.14	(0.46–3.07)	0.78
Ineffective ventilation	123	1.42	(0.74–2.69)	0.29	1.31	(0.72–2.68)	0.43
Existing airway obstruction	25	0.74	(0.16–3.32)	0.69	0.81	(0.20–4.10)	0.79
Impending airway obstruction	79	1.49	(0.71–3.13)	0.29	1.41	(0.68–3.13)	0.37
Combative or uncooperative	101	0.30	(0.07–1.22)	0.09	0.28	(0.07–1.19)	0.08
Relief of pain or distress	46	1.19	(0.40–3.56)	0.76	1.03	(0.35–3.29)	0.96

^a^In decades, centred on 50 years (e.g., an ‘age’ of 1.3 equals 50 + 1.3 × 10 years = 63 years). See Fig. 2 for a graphical representation of the estimated age effect

^b^Including drowning and asphyxia

^c^Including burns and strangulation

### Complications

Problems and complications recognised on-scene following attempted intubations were recorded in 13 % of the 2,144 patients, with recognised oesophageal intubation being the most frequent (25 % of patients with complications). Eight patients, seven in CA, suffered unrecognised oesophageal intubation by ground ambulance paramedics prior to HEMS arrival. All were subsequently recognised and reintubated by HEMS on-scene.

Complications related to the number of TI attempts in non-CA patients are shown in Table 3. There were more vomit/aspirations in the CA group (odds ratio: 2.1; 95 % CI: 1.3–3.5; *p* = .007), but no other statistically significant differences in complication rates between CA and non-CA patients (results not shown).

**Table 3:** Complications following intubation attempts of non-cardiac arrest patients

Attempts at airway intervention										
	One attempt *n* = 1,159		Multiple attempts by one provider *n* = 85		Multiple attempts by two or more providers *n* = 45		Not successful *n* = 9		Total *n* = 1,298	
Oesophageal intubation	0	0.0 %	17	20.0 %	9	20.0 %	4	44.4 %	30	2.3 %
Right bronchus intubation	4	0.3 %	2	2.4 %	0	0.0 %	0	0.0 %	6	0.5 %
Dental trauma	1	0.1 %	0	0.0 %	0	0.0 %	0	0.0 %	1	0.1 %
Vomiting and/or aspiration	17	1.5 %	4	4.7 %	1	2.2 %	0	0.0 %	22	1.7 %
Hypoxia	24	2.1 %	8	9.4 %	10	22.2 %	2	22.2 %	44	3.4 %
Bradycardia	7	0.6 %	1	1.2 %	2	4.4 %	0	0.0 %	10	0.8 %
Hypotension	35	3.0 %	1	1.2 %	3	6.7 %	1	11.1 %	40	3.1 %
Other^a^	18	1.6 %	8	9.4 %	3	6.7 %	0	0.0 %	29	2.2 %
None	1,063	91.7 %	51	60.0 %	23	51.1 %	4	44.4 %	1,141	87.9 %

Airway-related complications were defined as such if they were *not* present before the airway intervention and were recorded during or immediately after the airway management. It was possible to record more than one complication per patient

^a^Other complications, e.g. technical problems like laryngoscope failure, tube cuff damage, minor bleeding or accidental extubation

## Discussion

This study is the first prospective multicentre study to collect and compare advanced airway management data across international physician-staffed HEMS using a uniform template for data reporting. Our results show a 14.5 % risk of failure on first intubation attempt, but the consequences of failure were probably minor, as all airways were handled proficiently with a subsequent successful TI or an alternative airway approach. Also, our results indicate major differences in airway management between CA and non-CA patients, and identifies important risk predictors for first-attempt TI failure in the field.

Prehospital TI cannot automatically be compared to TIs performed in the emergency department or in the operating theatre, for two main reasons. Firstly, the majority of prehospital TIs are done in CA patients or after major trauma in challenging settings, while the majority of in-hospital TIs are done in a controlled environment. Secondly, prehospital TIs are challenged by a number of environmental factors that may influence the failure rates and increase adverse events [1, 2]. Restricted patient access, suboptimal patient and operator positioning, limited equipment and difficult or hazardous operating environments may increase prehospital intubation failure rates [12, 13]. Thus, the reported incidence of unanticipated difficult airways, first attempt failure rates, and rate of complications are higher in emergency TIs [5, 14, 15]. In our study, both overall and first attempt failure rates were comparable to other studies describing airway management in physician-staffed HEMS [16, 17].

Despite very low overall TI failure rates in both CA and non-CA patients, we found significantly higher first attempt intubation failure rates in the CA group. More vomit/aspirations found in these patients may partly explain the increased first attempt failure rates. Attempting TI during ongoing resuscitation efforts may also be challenging, and offers less opportunity to plan the airway management, due to competing priorities such as chest compressions being performed simultaneously. In order to minimise ‘hands-off time’, TI is often performed during chest compressions with the patient positioned on the floor or on the ground. In contrast, intubating trauma or non-CA medical patients on half-height ambulance stretchers with 360-degree access, an approach adopted by many services, allows for better TI conditions. Although the majority of the CA patients received TI on-scene, most were ‘cold intubations’ without the need of drugs, reflecting current practice in most services dealing with out-of-hospital cardiac arrests. However, a small group of CA patients may have intact airway reflexes and agonal respiration due to good quality cardio-pulmonary resuscitation and sustained circulation of the brain stem, presenting the need for NMBA or analgesics/sedatives to optimise TI conditions.

Prehospital drug-facilitated TI of non-CA trauma and medical patients may occur in less optimal conditions. As repeated attempts to facilitate TI may significantly increase the rate of adverse airway and haemodynamic effects, preoxygenation to avoid procedural hypoxaemia is recommended [18]. Although preoxygenation was not a recorded variable, it is standard operating procedure in most services for prehospital RSI [8]. Spontaneously breathing patients may preoxygenate adequately on non-rebreather masks, prior to RSI. The recorded first airway intervention for non-CA patients was immediate TI in three out of four patients. However, using the template definitions, it proved difficult in some cases to gather compliant data from ground ambulance services to ascertain what kind of airway management was performed by ambulance personnel prior to HEMS-physicians arrival on-scene.

Regarding drugs used to facilitate airway management, the majority of non-CA patients received standard RSI using analgesics, sedatives and NMBA, or an anaesthetic agent (e.g. ketamine) and NMBA. The rest were intubated with combinations of analgesics/NMBA or sedatives/NMBA, or, rarely, NMBA only, suggesting a degree of variation likely related to patient condition or provider preference. The more cautious use of traditional anaesthesia may be due to patients having decreased level of consciousness or being assessed as being circulatory unstable on-scene. The vast majority of non-CA patients received NMBA, which is recommended for optimising TI conditions and to decrease failure rates and complications. The rate of airway-related complications is comparable with those of other studies, and the association between an increasing rate of adverse effects following increasing number of TI attempts remains valid also for our study [19]. Also, our findings support the notion that prehospital airway management has more in common with emergency department RSI than elective anaesthesia, and may require a different approach to training and skill proficiency [14].

Our models seem to have good predictive power (Table 4), and the results suggest that the patient’s age was an important risk predictor for intubation failure, peaking at around 50–60 years before declining again (Fig. 2). This remained true when adjusted for other factors, such as patient category, intubation experience and the indication for airway management. For instance, a 50-year old patient had an estimated 60 % increased odds of having a failed first TI attempt compared to a 30-year old. The association between (increasing) patient age and difficult TI has previously been shown for some patients groups undergoing elective surgery, but is, to our knowledge, a novel finding in prehospital emergency TIs [20, 21]. A possible explanation for this age association is reduced head and neck movement, reduced thyromental distance and inter-incisor gap, along with worsening dental status in older patients [20].

**Table 4:** Model-based and empirical mean risks for first-attempt intubation failure, classified by model-based risk deciles (*n* = 1,200)

	Decile									
	1	2	3	4	5	6	7	8	9	10
Model-based risk	0.01	0.02	0.03	0.04	0.06	0.08	0.11	0.14	0.21	0.35
Empirical risk	0.04	0.02	0.01	0.07	0.07	0.12	0.07	0.16	0.25	0.26

The model-based risks are based on 10-fold cross-validation predictions from the mixed-effects logistic risk model for first-attempt intubation failure, using estimated conditional modes for the random effects

We agree with a Cochrane review suggesting that competence may be a key issue in emergency TIs, and non-physician-staffed services have shown higher TI failure rates [4, 22]. Although the majority of TIs in our study were done by experienced providers, some services allowed HEMS flight paramedics to intubate some patients under local SOP with physician supervision. Using the most experienced group as reference value, we could not show any significant association between failure rates and the level of prior TI experience. Supervision by experienced physicians during emergency TI has been reported to significantly reduce the complication rate, and our study suggests that the presence of experienced prehospital physicians on-scene may be beneficial [23]. Acknowledging the potential for harm in failed prehospital TIs, our results suggest that this may be minimised by experienced HEMS-physicians with high airway management proficiency, capable of detecting and correcting errors quickly, along with working backup plans and regular airway management training [22].

Although rarely performed, rescue procedures such as surgical airways may be the logical last step in prehospital difficult airway/failure to intubate patients, e.g. massive maxillofacial trauma (primary airway) or in *cannot intubate / cannot ventilate* settings (secondary airway). This emphasises the need for robust selection and training programmes for physicians working in prehospital HEMS systems. The surgical airway rate in our study (0.3 %) is low [17]. This is likely due to the implementation of robust difficult airway or RSI standard operating procedures in most services, employing a SAD as a rescue device before surgical airway in failed TIs, which is normally faster and easier [24].

Nearly three out of four patients included in our study were male, and while this is slightly higher than described in other studies, sex asymmetry in prehospital trauma and CA is expected [19, 25]. The question remains whether this translates to outcome differences. Although Rose and Cohen identified an increased risk for difficult TI in male patients receiving general anaesthesia, we could not confirm this in our study [21].

The strength of this observational study is in the prospective design and the use of a uniform template for airway data reporting, allowing high-quality research data to be compared across international HEMS systems and patient populations. Using the template in physician-staffed services, and monitored by local project coordinators, proved a reasonably robust system. Standardised and predefined variables can enhance the quality of data reported, as data normally collected for other purposes may be associated with uncontrolled operator or selection biases [26]. We believe the approach used in this study increases the level of evidence in airway research. Our results may not generalise to paramedic-staffed services, but as this study includes 2,327 patients from twenty-one HEMSs in six countries, we believe our results can be generalised to other HEMSs in developed countries, and we believe they may be useful for other physician-staffed services.

One of the main limitations was that recording of data was done by the treating physicians, with the risk of registration or recall bias. There is also a risk of clinicians underreporting adverse events or problems performing TI. Using anonymous forms in this study may have reduced this effect. Also, the Utstein-style airway template consists of nearly 50 variables to be registered per patient, and this level of detail may lead to registration fatigue, errors and missing data. Inter-observer reliability testing was not done before implementing the template into clinical service. The competence level of the assistant may influence the rate of TI success and complications, but we did not collect data on this. Different service-specific TI protocols, medications and culture of airway training are also factors that may influence the results.

We believe that future studies should examine the differences between in-hospital and prehospital emergency TI, concentrating also on the effect of prehospital intubation and post-intubation management on clinical outcomes. Recognising that prehospital TI is a complex intervention, improved study designs may be needed to link the effect of data from prehospital airway management to in-hospital outcomes [3]. The Utstein-style airway template should be revised further, for instance by limiting the number of variables that are difficult to collect in clinical studies, and increase precision level regarding performance of the airway intervention itself to include factors like RSI and preoxygenation. This may improve the dataset definitions towards a final airway data template.

## Conclusions

Advanced airway management in physician-staffed prehospital services was performed frequently, with high intubation success rates and low complication rates overall. However, cardiac arrest patients showed significantly higher first-attempt failure rates compared to non-cardiac arrest patients. All failed intubations were handled successfully with a rescue device or surgical airway.

## Abbreviations

TI: Tracheal Intubation
HEMS: Helicopter Emergency Medical Services
CA: Cardiac Arrest
STROBE: Strengthening the Reporting of Observational studies in Epidemiology
RSI: Rapid Sequence Induction
SOP: Standard Operating Procedures
NMBA: Neuromuscular Blocking Agents
SAD: Supraglottic Airway Device
BVM: Bag-Valve-Mask Ventilation
ETCO_2_: End Tidal Carbon Dioxide
CPAP: Continuous Positive Airway Pressure
